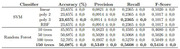# Analysis of machine learning algorithms to aid in the diagnosis of early‐stage dementia

**DOI:** 10.1002/alz.095187

**Published:** 2025-01-09

**Authors:** Thiago Carvalho, Maira Santana

**Affiliations:** ^1^ Universidade Federal de Pernambuco, Recife, Pernambuco Brazil

## Abstract

**Background:**

Dementia, including Alzheimer’s disease, represents a growing challenge for public health, with early diagnosis being crucial to delay or even prevent progression to more severe forms of dementia, allowing individuals to maintain a healthy life for as long as possible. It also enables patients and their families to plan ahead for future care and needs. However, diagnosing in the pre‐dementia phase is quite challenging, as early symptoms can be subtle, and there is no single test that can definitively diagnose the disease; diagnosis is made through a combination of clinical evaluation, cognitive tests, and imaging exams. The use of machine learning algorithms emerges as a promising approach to identify diagnoses of mild cognitive impairment (MCI), offering the ability to identify complex patterns in patient data that may not be easily perceptible to healthcare professionals. Therefore, the aim of this study is to assess the performance of artificial intelligence algorithms in identifying early‐stage dementia from slices of magnetic resonance imaging represented by shape and texture features.

**Method:**

To this end, an experiment was conducted using the Random Forest and Support Vector Machine (SVM) classification algorithms, carried out on a database composed of magnetic resonance images of 984 individuals, divided into three classes: Alzheimer’s disease, Mild Cognitive Impairment and Normal Cognition, with the intention of classifying among these three classes and attempting to predict which one a brain image would belong to.

**Result:**

The results of the experiment revealed that SVM did not perform well, achieving a maximum accuracy of only 23.65%, regardless of the configuration used. On the other hand, Random Forest demonstrated promising performance, reaching a maximum accuracy of 56.08% with 150 trees.

**Conclusion:**

However, it is important to note that despite the progress made, there are still significant challenges to be overcome in the quest for accurate and reliable methods for early diagnosis of dementia through magnetic resonance imaging.